# Retinal Organoids and Retinal Prostheses: An Overview

**DOI:** 10.3390/ijms23062922

**Published:** 2022-03-08

**Authors:** Alessandro Bellapianta, Ana Cetkovic, Matthias Bolz, Ahmad Salti

**Affiliations:** Center for Medical Research, Faculty of Medicine, University Clinic for Ophthalmology and Optometry, Johannes Kepler University Linz, 4020 Linz, Austria; alessandro.bellapianta@jku.at (A.B.); ana.cetkovic@jku.at (A.C.); matthias.bolz@jku.at (M.B.)

**Keywords:** retinal dystrophy, retinal organoids, iPSCs, 3D models, retinal prosthesis, photovoltaic polymers, organic semiconductors, blindness, restore vision

## Abstract

Despite the progress of modern medicine in the last decades, millions of people diagnosed with retinal dystrophies (RDs), such as retinitis pigmentosa, or age-related diseases, such as age-related macular degeneration, are suffering from severe visual impairment or even legal blindness. On the one hand, the reprogramming of somatic cells into induced pluripotent stem cells (iPSCs) and the progress of three-dimensional (3D) retinal organoids (ROs) technology provide a great opportunity to study, understand, and even treat retinal diseases. On the other hand, research advances in the field of electronic retinal prosthesis using inorganic photovoltaic polymers and the emergence of organic semiconductors represent an encouraging therapeutical strategy to restore vision to patients at the late onset of the disease. This review will provide an overview of the latest advancement in both fields. We first describe the retina and the photoreceptors, briefly mention the most used RD animal models, then focus on the latest RO differentiation protocols, carry out an overview of the current technology on inorganic and organic retinal prostheses to restore vision, and finally summarize the potential utility and applications of ROs.

## 1. Introduction

The eye is one of the most important sensory organs in humans, providing us with a valuable remote sense, vision. A wealth of information enters the visual system through the eyes, creating complex images with shapes, colors, and textures [1]. The majority of our perceptions of the world, as well as our memories of it, are based on this perceptual modality. As optically oriented beings, humans use this sense to image the environment on the retina, which is the light-sensitive, information-processing part of the eye [1,2]. Impairment at various stages of image- or information-processing can result in a wide variety of eye disorders ranging from restorable to non-correctable unilateral or bilateral vision loss, significantly reducing the quality of life [3]. Many forms of vision loss, such as age-related macular degeneration (AMD), retinitis pigmentosa (RP), glaucoma, and diabetic retinopathy (DR), involve the death of retinal cells that are critical for vision [4]. As life expectancy has increased and the global population rises, these eye disorders become more prevalent. 

Inherited retinal dystrophies (IRDs) are a class of disorders marked by the gradual degeneration of photoreceptors (PRs), resulting in vision loss and subsequent blindness. The incidence of IRDs is one case in every 3000 people. IRDs can be classified as cone, rod-cone, or cone-rod dystrophies, depending on which photoreceptor is initially impaired. This family of disorders has a broad clinical spectrum and a large number of implicated genes, with 271 genes for both syndromic and non-syndromic retinal dystrophies, resulting in significant clinical and genetic variability [5]. The most frequent types of IRDs, such as retinitis pigmentosa, Leber congenital amaurosis (LCA), inherited macular degeneration (MD), cone-rod dystrophy (CRD), Stargardt disease, congenital stationary night blindness (CSNB), enhanced S-cone syndrome (ESCS), and Usher syndrome (USH), may show overlapping clinical symptoms and genetic mutations [6]. 

Despite the advances in understanding retinal development and the preclinical translation of potential gene or cell replacement therapies, to date no treatments or definitive cures are available to reverse the degenerative processes of retinal dystrophies (RDs) or to restore vision [7,8]. Cellular and animal experimental models have been widely used to dissect the mechanisms involved in retinal diseases [9,10]. However, interspecies eye differences such as morphology, functionality, physiology, and molecular profile in animal models make the comparison and extrapolation to humans difficult [9,11]. The reprogramming of somatic cells into induced pluripotent stem cells (iPSCs) and the breakthroughs in their differentiation into three dimensional (3D) retinal organoids (ROs) have greatly improved the studies to treat RDs [11,12]. In parallel, research advances in the field of electronical retinal prosthesis using inorganic photovoltaic polymers have provided a promising opportunity in partial vision restoration [13,14,15]. Recently, the emergence of organic semiconductor polymers represents an encouraging alternative for retinal prosthesis, because they possess key prosthetic features such as photo-stimulation characteristics, flexibility, and biocompatibility [16,17,18]. In this review, we will focus on the latest advancement in stem-cell-derived RO protocols, summarize the current status of inorganic and organic retinal prosthesis to restore vision, and discuss the potential utility of ROs in RDs.

## 2. General Information about the Retina

### 2.1. Retinal Cell Population

**Figure 1:**
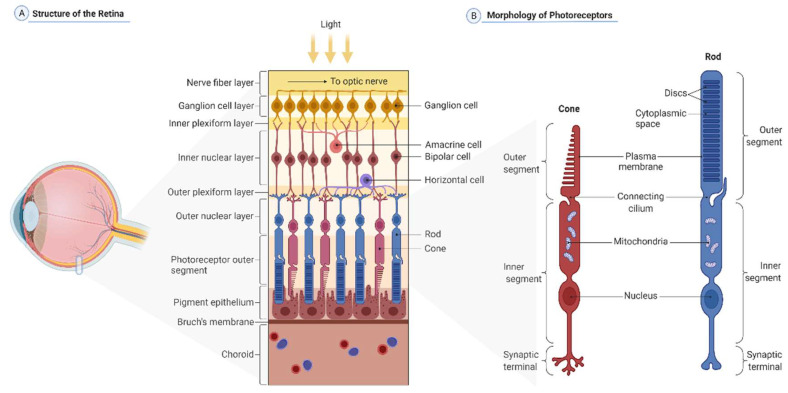
(**A**) Cellular organization of the retina. The retina contains the retinal neuronal cell types, such as the retinal pigment epithelium (RPE), which faces choroidal blood vessels at the basal side, and cones (purple) and rods (blue) at the apical side. The photoreceptor nuclei constitute a layer called the outer nuclear layer (ONL), whereas their axons and processes meet with horizontal (violet) and bipolar (red) cells in the outer plexiform layer (OPL). More anterior, the inner nuclear layer (INL) harbors nuclei of the bipolar (red), amacrine (pink), and horizontal (violet) cells, and Müller glia, while the inner plexiform layer (IPL) contains the processes and synapses of bipolar (red) cells, amacrine (pink) cells, and retinal ganglion cells or RGCs that are reduced in number by the stage of photoreceptor maturation (yellow). (**B**) Structure of rod and cone photoreceptors. Photoreceptors are polarized sensory neurons. Rods (blue) and cones (red) have three cellular compartments. Outer segments (OS) are stacks of membrane disks rich in the visual pigment rhodopsin. This is where phototransduction originates. Interestingly, this cellular part does not contain any protein synthesis machinery. All OS proteins are synthesized in the inner segments (IS) and then transported to this cellular part. IS also contain other vital organelles, i.e., mitochondria, and the nucleus. Neuronal impulses created in the OS pass through the IS until they reach the synaptic terminals, where they are transmitted to other retinal neurons. (Created with BioRender.com).

Prior to reviewing the latest research in the field, it’s crucial to understand the various cell populations that characterize the human retina (Figure 1A). The retina is a circular plane layer of tissue that coats the back of the eye. It extends from the center, the area centralis, to the ora serrata, the rim transition zone. The circular retina sheet spans 30–40 mm in diameter, while the retina itself is approximately 0.5 mm thick [19]. Based on embryonic development, it can be distinguished into two layers: an inner neural retina comprising retinal neurons and glia, and an outermost epithelial layer of cells containing pigmented melanosomes, which are referred to as retinal pigmented epithelia (RPE) cells [20]. The neural retina is composed of six basic types of retinal neurons with numerous subtypes and glia cells, the most prominent of which are the Müller glia, and can be further subdivided into distinct layers [19]. Since the neural retina is inversely composed, light must pass through the entire retina before being detected by two types of PRs at the outermost layer, the rods and the cones (Figure 1B). Their cell bodies are located in the outer nuclear layer, whereas their apical processes, the inner and outer segments, are in a distinct segment layer. The outer limiting membrane is a tight connection belt that separates the two layers. PRs create synapses with interneurons that are located in the inner nuclear layer, forming the outer plexiform layer. These neurons—bipolar cells, horizontal cells, and amacrine cells—are responsible for the initial processing of light-induced stimuli. Finally, the bipolar cells transmit the processed information to the ganglion cells. Synapses between the bipolar and the ganglion cells are present in an inner plexiform layer, whereas ganglion cells’ nuclear bodies establish a distinct ganglion layer. The axons of the ganglion cells, which are located in the innermost layer of the retina, are then condensed to the optic nerve, which eventually projects to the visual cortex in the brain. Astrocytes occupy the most anterior layer constituting, with Müller cells end-feet, an inner limiting membrane that separates the retina from the vitreous. 

### 2.2. Photoreceptors

The average human retina harbors approximately 92 million rod PRs and 4.6 million cone PRs [21]. Their distribution pattern is rigorously regulated by a predominance of rods in the periphery and a dense packing of cones primarily in the retina’s center, the fovea centralis. It is the field with the highest resolution because it has a one-to-one linkage to bipolar cells and is the focus of the refracted light [19,22]. Both rods and cones have the same basic structure (Figure 1B), and they both have an inner and outer segment with a connecting cilium [23] and a distal end-foot that forms the terminal synapse connecting the PRs to the interneurons [24]. The inner segments of both PR types contain a significant amount of endoplasmic reticulum, ribosomes, Golgi apparatus, and a multitude of mitochondria right next to the outer segment [23,25]. The outer segments are made up of stacked disks of membranes that hold the chromophores, rhodopsin in rods and L/M/S-opsins in cones [26]. Disks are formed as protrusions of the outer segment membrane towards the connecting cilium at the base, whereas at the apex, they are shed off and phagocytosed by RPE cells [27], allowing for recurring regeneration of outer segment disks. Rod PRs respond most strongly to 500 nm light waves due to the expression of the light-sensitive pigment rhodopsin [23,28]. The rod cell is very sensitive to a small number of photons and thus crucial for vision in low-light circumstances [29,30]. Cones are categorized into three subtypes based on their ability to detect a certain light spectrum: short-wave (s) cones, which detect blue light with a maximum wavelength of 420 nm; middle-wave (m) cones, which detect green light (530 nm); and long-wave (l) cones, which detect red light (560 nm) [31]. The distinction in light wave detection is linked to the expression of specialized opsins (S-, M-, and L-opsin) and allows for color vision and discrimination [32]. 

## 3. Basics of Eye Development 

The retina, RPE cells, and the margin are the three origins of the eye structures in vertebrates. The iris epithelium is derived from the neuroectoderm, the lens is formed from lens placode, and the extraocular mesenchyme is fabricated from neural crest cells and mesoderm [20]. The emergence of two eye fields bilaterally evaginating from the neuroepithelium of the ventral forebrain, generating the so-called optic vesicles, is the first step in the development of the eye. The genesis of the eye is driven by a coordinated change in cell shape and behavior, which is regulated by transcription factors such as *Pax6*, *Rax*, *SIX3*, *LHX2*, *SIX6*, and *OTX2* [33,34,35]. The optic fissures will be formed later by the optic stalk, which connects the optic vesicles to the neural tube. In tandem with the optic vesicle, the lens placode is initiated by the evagination of the neighboring surface ectoderm. This lens placode invaginates into the direction of the optic vesicle in a second stage. As a result, the central region of the optic vesicle begins to invaginate, forming an optic cup. At this point, the retinal progenitors in the vesicle’s dorsal region commit to the fate of RPE cells [20,36,37]. The main signaling molecules influencing the developmental fate of the optic cup and optic vesicles include *WNT*, *BMP*, *FGF*, and *SHH* [38]. The neural retina is formed by ventral progenitors, which later give rise to all retinal neurons and glial cells. *Vsx2* (*Chx10*) and *MiTF* are the critical factors that trigger these commitments. *MiTF* is initially expressed in all retinal progenitors in mice and is downregulated by Vsx2 expression. The remaining MiTF+ cells generate the RPE when the Vsx+ progenitors commit to a neuronal retina fate. Later, *MiTF* plays an important role in the upregulation of terminal RPE differentiation and pigmentation genes such *Dct*, *Trp1*, and Tyrosinase [37,39,40]. Although the cellular mechanism of the optic cup infolding is unclear, contractile filopodia might provide a mechanical force driving the process [41].

## 4. Animal Models

Significant progress has been made in recent years in understanding retinal diseases and identifying potential therapeutic targets for intervention [4]. Different animal models are often used in biomedical research, ranging from naturally occurring to genetically engineered and from insects to mammals. Rodents are by far the most-used animal models due their relatively low cost, short reproduction cycles, and the opportunities for genetic manipulation to generate knock-out (KO) and knock-in (KI) strains [42,43,44]. A summary of the most-used rodent animal models of RDs is presented in Table 1. The first reported animal model for RDs was a rodless mouse, named rd1 mouse, which is the most commonly used mouse model for RP, featuring PRs cell death. For AMD, *Nrf-2*, *SOD1* or complements *H, C3a, C5a* KO mice or mice expressing the human *ApoE4* or *C3* genes, are usually used. These models recreate some of AMD’s pathological hallmarks, such as choroidal neovascularization and thickening of Bruch’s membrane. The chemokine signaling *Ccl2/Cx3cr1* deficient mice is also described to exhibit AMD-like features. For glaucoma, the most commonly used model is the ocular hypertension TDBA/2J mouse strain due to the spontaneous, age-related degeneration of retinal ganglion cells (RGCs). For diabetic retinopathy, genetic diabetes models are usually used, such as the Ins2Akita with a mutation in the insulin-2 gene or the ob/ob mice carrying a mutation in the leptin gene [42,45]. 

Nevertheless, large animal models have significant benefits over normally employed laboratory mouse models. Their globe size and dimensions are more similar to those of humans, and they feature a retinal zone with a high cone density and denser PRs packing for high acuity vision. Porcine eyes and retinae are increasingly used for studying human retinal diseases owing to the comparable anatomical features with the human situation in terms of size, general morphology, vascularization, and the simplicity with which transgenic animals can be produced [42,46]. Since spontaneous IRDs are frequent in the canine pet population, the dog is the most commonly used species. Cats can also develop IRDs on their own. Sheep, horses, and non-human primates are other notable animal models with spontaneous IRDs. Current work is in progress to generate engineered models of other major animal species, including non-human primates [47]. 

Despite their benefits, retinal disease animal models rarely represent the full human disease situation. The reason for this limitation is due to species differences with regard to retinal anatomy, PRs types, or genomic conservation compared to humans, along with ethical constraints. In vitro models are becoming a more popular alternative to animal models [48]. iPSCs derived from an individual’s somatic cells, for example, can be genetically reprogrammed to become pluripotent, which means they can differentiate into any other adult cell type [49]. Differentiated cells derived from these iPSCs contain the individual’s unique genome and, as a result, have the exact genetic and cellular context to study disease mechanisms of hereditary disorders. Stem cells offer new opportunities for modeling retinal diseases, and stem cell-derived 3D ROs have recently become the focus for therapeutics and disease research.

## 5. Retinal Organoid Protocols

Organoids are 3D in vitro replicas of specific organs that comprise several organ-specific cell types with a spatial configuration that resembles the natural tissue [50]. So far, differentiation protocols for diverse organoid types exist, including brain organoids [51,52,53], intestinal organoids [54,55], lung organoids [56], and kidney organoids [57].

### 5.1. Two-Dimensional (2D) Retinal Differentiation

In the 2000s, the possibility of culturing human embryonic stem cells (hESCs) in vitro and the discovery of iPSCs paved the way for the generation and differentiation of neural tissues, including retinal cells [58,59]. One of the earliest successful differentiation strategies directed ESCs towards an anterior neural fate using adherent two-dimensional (2D) cultures [60,61,62]. The addition of Wnt/BMP signaling inhibitors to the medium, together with IGF-1, promoted the development of PR-marker positive cells, but these were not the predominant cell types in the adherent culture [62]. The addition of the rod-genesis factors retinoic acid (RA) and taurine increased the number of PR-marker positive cells when Notch signaling was inhibited by DAPT treatment [61]. Neural induction media comprising heparin and a chemically defined N2 supplement stimulated iPSCs to assemble embryoid bodies, which then adhered to the surface of the coated culture dish and differentiated towards neural retina [63]. The number of PRs achieved under these conditions, however, was limited. These PRs were predominantly precursor cells scattered in a monolayer of mixed cultures.

### 5.2. Three-Dimensional (3D) Retinal Differentiation

The Sasai group pioneered organoid research in 2005, when they established a procedure for selectively differentiating murine ESCs to neurons [51,64]. In the years thereafter, the same group discovered ways to differentiate murine and then hESCs into optic cups [65,66]. These discoveries served as a foundation for other researchers to establish hiPSC-derived RO differentiation protocols [67,68,69,70]. The switch to non-adherent (3D) protocols was a critical step in obtaining stratified neural retinas. Table 2 recapitulates the key protocols used to date in generating 3D ROs. Mouse (m)ESC aggregates cultured in suspension under low-growth factor conditions, in combination with the Matrigel extracellular matrix, increased the development of optic cups with apical-basal polarities [65]. Eiraku and colleagues observed that these organoids could self-assemble an optic vesicle and, subsequently an optic cup, without the support of an external structure. The optic cups from mESCs, and later of hESCs, included neural retinal progenitors as well as RPE cells in the proper orientation. When ROs derived from hESCs, mESCs, or hiPSCs were differentiated into optic vesicles, the emergence of neural retinal neurons was also observed. All of the retinal cell types, as well as the inner and outer limiting membranes, were present. Furthermore, these organoids exhibited a primitive but accurate layering, with an exclusive PR nuclei layer (similar to the outer nuclei layer), an inner layer harboring interneuron, and an inner GCL [65]. The protocol adopted in a study conducted by Nakano and colleagues [66] was based primarily on a 3D culture of embryoid bodies that were under pro-neural, neural tube-inducing conditions and in the presence of growth factor-reduced Matrigel primed in an anterior forebrain direction. This was accomplished by inhibiting Wnt signaling and subsequently activating the hedgehog pathway. The use of fetal bovine serum and the hedgehog agonist SAG improved retinal differentiation in human stem cells with laminated retinas, which expressed markers for all retinal cell types: ganglion, amacrine, bipolar, horizontal, Müller, and PR cells. Electron microscopy investigation of the PR cell layer in human ESC-derived ROs revealed mitochondria and rudimentary connecting cilia with basal bodies, but no apparent outer segment [66].

### 5.3. 2D/3D Retinal Differentiation Technique

Zhong and colleagues used a sequential 3D and 2D cell culture as well as undirected neural differentiation to generate hiPSC-derived optic vesicles [67]. iPSCs were differentiated into mature and light-responsive PR cells with rudimentary outer segments using a combination of 3D and 2D protocols that did not require the inclusion of small molecules. This was accomplished by lowering the RA concentration between day 50 (d50) and d70 and extending the culturing timeframes. They observed that neural induced iPSCs had a proclivity to form forebrain and, in particular, diencephalon-derived retinal progenitors. When retinal progenitors were allowed to develop 3D spheroids, they formed the same organoids that Nakano and colleagues described. Based on their ability to form retinal neurons, photosensitive rod and cone PRs, appropriate layering, and PR ribbon synapses, these organoids are nearly indistinguishable [66,67]. A 2D to 3D technique, on the other hand, allowed the bypass of embryoid body formation, resulting in neuroretinal structures in adherent culture that were excised and cultured in suspension [72]. These floating neuroretinas generated neural rosettes with PRs but lacked the lamination seen in other 3D cultures. The addition of differentiating retinal factors—serum, RA, taurine, and the supplements N2 and B27—enabled the formation of PRs with rudimentary outer segments observable at the margins of the ROs [80]. Interestingly, a distinct protocol beyond the 2D and 3D models produced neuroretinas with mature PRs after the spontaneous attachment and spreading of epithelial structures known as cysts [74].

### 5.4. Protocol Improvement

The majority of the aforementioned methods use the same media components, but a change in timing and the addition of particular molecular cues improved the yield of the neuroretinal vesicles formed. Contraction of the actin-myosin cytoskeleton is a major effector of hESC mortality after cell dissociation, and disrupting this contraction by inhibiting Rho-associated kinase (ROCK) or myosin light chain kinase can substantially enhance cell survival [81,82]. ROCK inhibitor is therefore commonly used in differentiation processes based on dissociation–reaggregation [60,83,84]. ROCK inhibition has also been shown to have a neurogenic influence on stem cells. Bone morphogenetic proteins (BMPs) have a role in retinal dorsal/ventral patterning [85], and BMP4 is required for retinal specification in mice [86]. The addition of timed BMP4 treatment was proven to promote neuroretinal epithelia self-formation [77,87]. When IGF-1 was supplied to the media during the first three months of differentiation, it also aided in the development of 3D-laminated ROs [79,88]. However, the response to BMP4 and IGF-1 activation is iPSC line-and-differentiation method dependent [89]. Using 9-cis retinal instead of all-trans RA accelerated rod PR differentiation in organoid cultures, with increased rhodopsin expression and more mature mitochondrial morphology, is visible by d120 [78]. Thyroid hormone signaling modulation aided in controlling the fate of cone subtypes in ROs [90]. RGCs typically emerged between d40 and d50 after differentiation began. Encapsulating embryoid bodies in a 3D Matrigel droplet instead of growing them in suspension resulted in accelerated ganglion cell growth within d28 of differentiation [91]. In addition, controlling the generation of embryoid bodies using agarose microwell arrays, combined with a checkboard pattern dislodging technique after plating in 2D, strongly influenced the efficiency of ROs’ production [92]. 

### 5.5. Differentiation Phases of 3D Retinal Organoids

ROs’ differentiation phases can be defined in three stages. Organoids form a clear phase-bright outer neuroepithelial rim that comprises neural retina progenitors and RGCs in stage 1, with differentiation at around d30 to d50. RGCs are the first retinal cells to differentiate at around d50, but their numbers decline after that, presumably due to a lack of linkage to brain targets [67,92]. 

A recent study found that when retinal and brain organoids were merged at d50, RGCs demonstrated axonal extension and pathfinding into cortical neurons of brain organoids, as well as increased proliferation and decreased cell death, at d150 [93]. Organoids acquired a phase-dark center with a diminished bright rim in the second stage, at around d80–120, and early progenitors of cones and rods started to emerge. 

The outer rim was more evident in stage 3, between d120–d180, with hair-like or brush-border-like elements that coincided to the photoreceptor inner and outer segments [77]. At each stage of maturation, the differentiating retinal organoid expressed a distinct set of specific markers. PRs, for example, expressed the transcription factor *PAX6*, as well as the retinal progenitor cell factor *VSX2*, early in differentiation, followed by the photoreceptor precursor-specific transcription factor *CRX*, the early rod-specific marker *NRL*, and the mature cone and rod markers recoverin, L/M/S-opsins, and rhodopsin, respectively. 

PCARE protein staining from d120–d180 can be used to follow photoreceptor cilium and outer segment formation throughout retinal development [94]. A dark patch of RPE cells is often seen as part of the growing neural retinal vesicle, but not as a monolayer covering the neural retina. The retinal organoid cell populations were expected to be fully established between d210 and d260, and afterwards to decrease in complexity [77,92].

### 5.6. Brain Organoid-Derived Neural Retina

Eye and brain diseases are now known to be more linked than originally anticipated. The eye and the brain form as extensions of the forebrain diencephalic and telencephalic regions of the developing central nervous system (CNS) [95]. Neurodegenerative alterations and disease patterns have been observed in both brain and eye areas in studies of RDs, such as glaucoma [96]. Complex organoids have the potential to produce effective in vitro models of various human diseases when they correctly recreate retinal development, morphology, and maturation. As a result, the enhanced development of the hPSC-derived retinal-brain link via the optic nerve is identified as crucial for degenerative disease modeling. Brain organoids can mimic individual brain regions or whole cerebral areas, and both can occasionally generate ocular regions [50,52,97]. A recent paper published by Fernando and Lee reported a straightforward and inexpensive approach for developing the neural retina and cortical brain regions from confluent cultures of stem cells. The cortical organoids isolated and cultured in suspension conditions for maturation were proven to be transcriptionally comparable to organoids produced by other protocols and to the human fetal cortex. Further maturation of this complex organoid system revealed the formation of optic nerve-like structures linking retinal and brain organoids, which may aid in the analysis of neurological diseases of the eye and brain [98]. Another recent study carried out by Gabriel et al. aimed to reduce the complexity and the timeframe needed to generate ROs by demonstrating the creation of forebrain-associated bilateral optic vesicles with cellular variety and functional evidence using iPSC-derived human brain organoids. They reported, at around d30, the propension of brain organoids to attempt the generation of optic vesicles, which eventually evolved into visible structures over the course of 60 days. Those optic vesicle-containing brain organoids (OVB-organoids) contained the cellular components of a developing optic vesicle, such as primitive corneal epithelial and lens-like cells, retinal pigment epithelia, retinal progenitor cells, axon-like projections, and electrically active neuronal networks. Interestingly, Synapsin-1, CTIP-positive myelinated cortical neurons, and microglia were also observed in OVB-organoids [99]. The creation of optic nerve in complex organoid structures in vitro will help in the better understanding of the dynamics of retinogenesis and neurogenesis, which may subsequently be utilized to model a wide range of ocular neuropathies such as glaucoma. Importantly, these retinal-brain organoids promise to enhance organoid growth, such as the long-term survival and maturation of RGCs in ROs, since these cells will no longer be cut off from the cortex’s output.

### 5.7. Limitations of Retinal Organoids

Currently, RO protocols show heterogeneity across various lines and even within and across organoids [92,100].This might be due to the epigenetic memory from the initial somatic cell that may either promote or inhibit hiPSC differentiation toward a specific lineage [101]. Other challenges of ROs are the poor and variable maturation states of PRs and the absence of direct contact with RPE, which result in ROs showing low response to light stimulation [12]. In addition, the effect of aging that constitutes a reason for progressive neurodegeneration and late-onset RD diseases may not be reflected in the current RO protocols, even with a long culture time [11]. However, the continuous improvement of protocols and the establishment of new techniques, such as retina-on-chip and co-culture systems [102], as well as the possibility of inducing aging by overexpressing Progerin or telomere shortening [103,104] to improve the efficiency, reproducibility, and maturation of ROs, might alleviate some of these problems. Furthermore, unbiased omics, including proteome investigations, as well as rigorous neural activity measures, are required to establish organoid variability and functionality.

## 6. Retinal Prosthesis to Restore Vision

Gene therapy, stem cell therapy, optogenetics, non-invasive stimulation, and retinal prostheses provide promising results as new therapeutic approaches to vision restoration [4,105,106]. Most of these treatments are still in preclinical or clinical phases of development, whereas only the gene therapy for RPE65-linked RDs is currently commercially available [107]. However, researchers have been particularly investigating different possibilities for the development of retinal prostheses since the 1960s, due to their potential in vision restoration. Some retinal prostheses were FDA- and CE-approved and commercially available in the United States and Europe, but their manufacture was subsequently suspended [108,109,110,111]. 

### 6.1. Principle of Electronic Retinal Prosthesis

Retinal prostheses are designed to replace damaged or lost PRs of the degenerated retina and to restore vision. Such devices were implanted in patients diagnosed mostly with late-onset IRDs or age-related degenerative diseases, where damage or loss of most of the PRs is present in the outer layers of the retina, but the inner retinal neurons are nearly intact. [111,112]. The principle of retinal prosthesis is based on an introduction of a visual information by the stimulation of neurons in the inner retinal layers, which, despite some later degeneration, are still preserved and capable of transferring visual signals to the brain [4]. The entire mechanism of replacing the function of damaged or lost PRs in the outer layers of the retina is based on either direct electrical stimulation or photodiodes. Direct electrical stimulation requires an internal and an external system to direct the current, via wires or wirelessly, to the electrodes comprising the implant. The image captured by an external camera-based system is transformed by image-processing algorithms into an electrical signal and then sent to an internal system, and the electrodes are implanted in the retina [111,113]. On the other hand, in photovoltaic implants, the electrical stimulation is generated by signal transduction of an incident light by micro-photodiode arrays. Natural or infrared light can be used as a light source; the latter is preferred because any light perception by the remaining visual pathway is avoided. Most importantly, natural eye movement is preserved and, therefore, the normal perception of vision is preserved [114,115]. Furthermore, implanted retinal prostheses provide patients with the ability to see phosphenes, the spots or rings of light. The image quality perceived by patients is regulated by the number of electrodes or photodiodes on the implant, the type of stimulation, and the adjustment of grey tone levels. Retinal prostheses provide unique vision that certainly differs from natural vision; therefore, patients are often in need of a comprehensive understanding, additional training, and practice to achieve the optimized results [108].

### 6.2. Types of Electronic Retinal Prostheses

Based on a surgical approach, four different types of retinal prostheses were designed: epiretinal, subretinal, suprachoroidal, and intrascleral prostheses implanted on the retina, beneath the retina, in the suprachoroidal space, and within the pocket in the sclera, respectively [4,111,116]. Both epiretinal and subretinal prostheses offer an improved visual resolution that allows patients to read short words [114,117]. An example of an epiretinal device that generates stimulation at the position closest to the targeted RGCs is the Argus II System (Second Sight Medical Products, Sylmar, CA, USA) which is comprised of 60 platinum electrodes with a polyimide microelectrode array. Its efficiency was initially evaluated by in vitro experiments on isolated tiger salamander retina [118]. It was used in more than 350 patients worldwide, thus being the most widely implanted retinal prostheses in the world. Even though it obtained regulatory approval and was commercially available, its manufacture was suspended in early 2019 [109,117,119]. 

On the other hand, subretinal prostheses were developed to be intentionally implanted in the location closest to the degenerated photoreceptor cells, between the RPE and the PR layer [116]. The Alpha-AMS/IMS (Retina Implant AG, Reutlingen, Germany) is an example of such a device, with 1600 electrodes. Stimulation threshholds and their relation to electrode size were validated by recording the spiking of RGCs of mouse rd10 retinas [120]. This device received CE mark approval, but the commercialization of the product subsequently ceased [110]. PRIMA (Pixium Vision, Paris, France) is another subretinal prosthesis type, currently in the clinical phase of development as a treatment for age-related macular degeneration. It is a photovoltaic retinal implant consisting of 378 electrodes, which does not require an external video camera as is the case with epiretinal prostheses. The efficacy of a photovoltaic stimulation was validated by an in vitro experimental setup using multielectrode arrays on rat retina [115]. A Gen 2 suprachoroidal device (Bionic Vision Technologies, Melbourne, VIC, Australia) and an STS device (Osaka University, Japan) are further examples of retinal prostheses [113,121]. Furthermore, research in vision restoration is a fast-growing field and many more retinal implants are on the horizon. POLYRETINA represents a newly developed photovoltaic retinal prosthesis with improved characteristics: wide-field, high-density, and high-resolution [122]. Recently, a novel graphene electrode was proposed for use in retinal implants due to its advantageous properties for stimulation [123]. Moreover, wireless silicon photovoltaic implants are currently in development for AMD patients and already show promising results [124,125]. 

### 6.3. Limitations of Electronic Retinal Prostheses

The current state-of-the-art electronic prosthesis systems have a thickness of ~30 to ~70 µm in order to implant the prothesis properly in the subretinal space and avoid implantation surgery complications. Hence, the implantation of multiple small microchips has been suggested [126]. Nevertheless, the stability of multiple modules in the subretinal space over time needs to be investigated, as well as the ability of blind patients to recover their vision. In addition, classic electronics are optimized to work in dry conditions, and electrical interconnects as well as many inorganic materials may oxidize in a physiological setting, necessitating tightly sealed passivation and encapsulation. Moreover, the size of the restored field of view directly relates to the size of the implanted prosthesis’s contact to the retina. A retinal coverage of 3 mm in diameter is required to restore a visual field of 10 degrees. Tests on normal individuals under pixelated vision indicated that 30° of visual field could provide adequate mobility skills [127]. Retinal prostheses covering a large field of view must have two important features: being foldable to limit the scleral incision, and being conformable to remain in tight contact with the retina over its entire surface. The poor visual resolution, in combination with the small visual angle, were important factors indicating why two major companies with regulatory-approved prostheses (Retina Implant AG, Reutlingen, Germany, and Second Sight Medical, Sylmar, CA, USA) stopped the commercialization of implantable devices. Scar formation has been occasionally mentioned for the Alpha AMS implant [128], and the Argus II™ implant required a bulky retinal tack [129]. Although silicon-based neuroprosthetics have shown promising results, including reading capability, only one implant (PRIMA, Paris, France) is currently in clinical trial [130]. Alternative techniques are being explored, and various organic materials are being investigated as possible alternative candidates for organic prostheses. 

### 6.4. Organic Retinal Prostheses

One of the most appealing approaches currently being investigated is the use of organic materials for neuronal stimulation, which have demonstrated promising results in terms of performance as well as appropriate biocompatibility and flexibility. Different organic semiconducting polymers and pigments have been investigated and tested for their performance and efficacy in retinal stimulation, as well as for the molecular aspects of biocompatibility [18,131,132,133] (Table 3). The most widely investigated material in the field of organic electronics is undoubtedly the organic semiconducting polymer called poly(3,4 ethylenedioxythiophene)-poly(styrenesulfonate) or PEDOT:PSS. Its use in neuronal as well as retinal stimulation has been extensively studied [134,135]. In recent studies, there was a huge interest in combining PEDOT:PSS with another organic polymer, regioregular poly(3-hexylthiophene-2,5-diyl) or P3HT, to improve the efficiency in neurostimulation. A fully organic retinal prosthesis, used for vision restoration in a rat model of blindness, is composed of P3HT as a semiconductive layer, PEDOT:PSS as a conductive layer, and a silk fibroin as a passive substrate layer. This combination showed very promising results, especially in terms of its excellent functionality and high biocompatibility and stability [132,136,137]. However, the most successful combination for the direct photostimulation of neurons at the interface was accomplished by combining the P3HT and [6,6]-phenyl-C61-butyric acid methyl ester (PCBM), where P3HT behaves as an electron donor material while PCBM behaves as an electron acceptor [17,132,137,138]. 

Recently, a novel approach was proposed by Maya-Vetencourt et al. that used only P3HT as conjugated semiconducting polymer nanoparticles. They were injected subretinally, and evoked light stimulation of retinal neurons in a rat model of retinitis pigmentosa [106]. Another class of materials that can be utilized for neuronal stimulation are organic pigments and dyes. Indigos, quinacridones, and phthalocyanines proved to be suitable for the development of optoelectronic devices [139,140,141,142]. Additionally, N,N′-dimethyl perylene-3,4:9,10-tetracarboxylic diimide (PTCDI) is another organic pigment that proved to be highly efficient in direct optoelectronic retinal stimulation [18].

**Table 3:** Summary of the most-used organic materials in the field of organic retinal prostheses.

Organic Material	Configuration	Purpose	Cytotoxicity	Validation Models	Publication
P3HT	rrP3HT—el. donor PCBM—el. Acceptor	Neuronal stimulation	Propidium iodine/fluorescein diacetate staining assay and patch-clamp recordings	Primary culture of hippocampal neurons	Ghezzi et al., 2011 [137]
	Single (donor)-component P3HT film	Subretinal stimulation	Propidium iodine/fluorescein diacetate staining assay and patch-clamp recordings on primary culture of hippocampal neurons	Sprague–Dawley albino rat retinal explants	Ghezzi et al., 2013 [17]
	P3HT—el. donor N2200—el. Acceptor	Epiretinal stimulation	TUNEL assay	Embryonic chick retina	Gautam et al., 2014 [143]
	P3HT—semiconductive layer PEDOT:PSS—intermediate conductive layer Silk fibroin—substrate	Fully organic SILK-PEDOT:PSS-P3HT prosthesis	No Inflammation after 6 months in vivo	In vivo RCS rat models	Maya-Vetencourt et al., 2017 [136]
	PEDOT:PSS—anode P3HT:PCBM—Semiconductor layer Titanium—cathode PDMS—substrate material;	Foldable, wide-field epiretinal prosthesis POLYRETINA	XTT cell viability assay	Ex vivo explants from rd10 mouse model	Ferlauto et al., 2018 [138]
	Conjugated polymer nanoparticles P3HT on PET substrate	Liquid retinal prosthesis (subretinal injection)	No inflammation after 240 DPI	RCS rat retinal explants; In vivo RCS rat model	Maya-Vetencourt et al., 2020 [106]
PCBM	MEH-PPV—el. donor PCBM—el. Acceptor	Hybrid solid-liquid polymer photodiode	—	Photocurrent action spectrum measurements in cell culture medium working as a cathode	Antognazza et al., 2009 [144]
	PDPP3T—el. donor PCBM—el. Acceptor	Near-Infrared Tandem Organic Photodiodes	—	Pulsed NIR illumination in a physiological environment	Simone et al., 2018 [145]
	PEDOT:PSS—anode PCPDTBT (or P3HT):PC_60_BM—BHJ Titanium—cathode	NIR-sensitive foldable and photovoltaic wide-field epiretinal prosthesis nirPOLYRETINA	XTT cell viability assay	Ex vivo explants from rd10 mouse models	Airaghi Leccardi et al., 2020 [131]
PTCDI	H2Pc: p-type el. donor PTCDI—n-type el. Acceptor	Epiretinal stimulation	—	Embryonic chicken retina	Rand et al., 2018 [18]

P3HT (poly(3-hexylthiophene-2,5-diyl); PCBM ([6,6]-phenyl-C61-butyric acid methyl ester); PTCDI (N,N′-dimethyl perylene-3,4:9,10-tetracarboxylic diimide); PEDOT:PSS (poly(3,4 ethylenedioxythiophene)-poly(styrenesulfonate)); MEH-PPV (poly[2-methoxy-5-(2’-ethylhexyloxy)-1,4-phenylene vinylene); PCPDTBT (poly[2,6-(4,4-bis-(2-ethylhexyl)-4H-cyclopenta [2,1-b;3,4-b′]dithiophene)-alt-4,7(2,1,3-benzothiadiazole)]); N2200 (poly{[N,N ′ -bis(2-octyldodecyl)-naphthalene-1,4,5,8-bis(dicarboximide)-2,6-diyl]-alt-5,5′-(2,2 ′-bithiophene)}); PDMS (poly(dimethylsiloxane)); PDPP3T (poly[[2,5-bis(2-hexyldecyl)-2,3,5,6-tetrahydro-3,6-dioxopyrrolo[3,4-c]pyrrole-1,4-diyl]-alt-[2,2′:5′,2′′-terthiophene]-5,5′′-diyl]); BHJ (bulk heterojunction); H2Pc (metal-free phthalocyanine); RCS (the Royal College of Surgeons) rat model (rd10, retinal degeneration 10 mouse model); NIR (near infrared); DPI, days post-implantation.

## 7. Utility of Retinal Organoids

**Figure 2:**
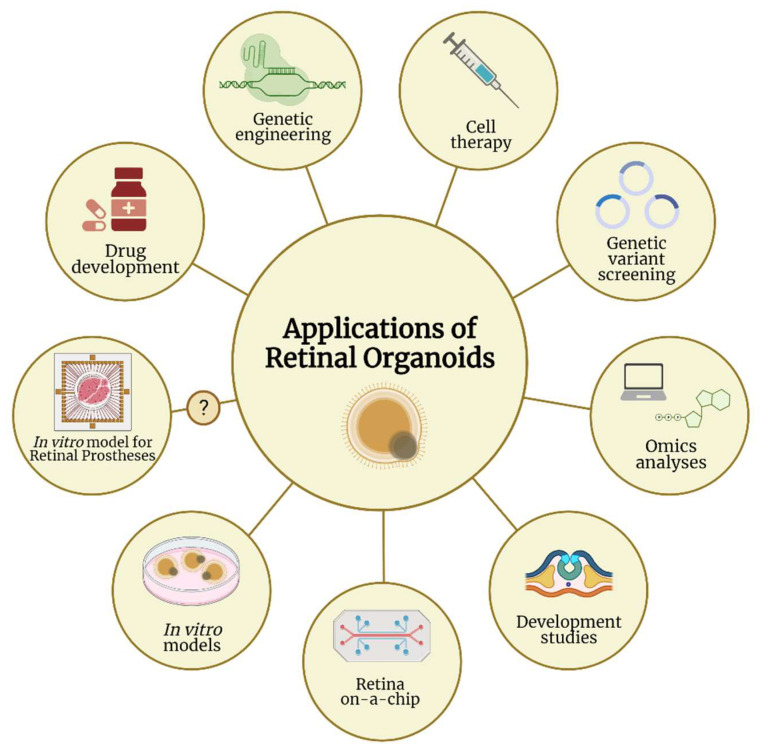
Applications of retinal organoids in various research fields. Retinal organoids carry great potential to be utilized in many research areas, from genetic engineering, omics analyses, and drug development to developmental studies and cell therapy. Retinal organoids can also be used as human in vitro models and in the recent emergence of retina-on-a-chip technology. One additional potential utility is to test the efficiency of retinal prostheses, when retinal organoids are used as human in vitro models recapitulating the disease pathophysiology. Despite few studies proving that retinal organoids are light-responsive on electrode arrays, this application on retinal prostheses has not yet been addressed in any publication, which justifies the question mark in the figure. (Created with Biorender.com).

Organoids as a source of human retinal tissue in vitro represent a great opportunity for various research and therapeutical applications. A schematic representation of the most important applications of ROs is shown in Figure 2.

### 7.1. Cell Replacement and Gene Therapies

One of the potential uses of human PSC-derived retinas is as a tissue source for retinal cell replacement therapy for blindness conditions, such RP, AMD, and glaucoma [146,147,148]. The key concern of cell replacement is to transplant and functionally integrate cells capable of developing into mature PRs, to restore the defective retinal tissue. Currently, two techniques of PR restoration are being pursued: transplantation of dissociated cells and transplantation of “sheets” of embryonic retinal tissue. Each of those techniques showed potential in animal models and even in a human clinical trial for retinal sheets [149,150]. Despite this intricacy, the PSC-derived retina experiments mentioned above showed that PSC-derived differentiating tissues have a remarkable capacity to organize and self-pattern [149,151]. Nonetheless, taking into account the diseased retina environment may be essential for successful transplantation because the cytoarchitectural remodeling of inner retinal neurons, namely gliosis, and neural retinal thinning at late stages may impair transplanted cells or the tissue’s ability to restore the precise neural circuitry that underpins the visual signals exiting the retina [151,152]. As a result, optimal functional restoration may need pre- or post-transplantation retinal remodeling.

ROs are a very new and quickly developing technology. They can be used to investigate gene therapy for a variety of retinopathies, such as by examining gene delivery in a human system. Therapies based on adeno-associated virus (AAV) vectors are gaining momentum as a potential treatment for retinal diseases. One major reason is the accessibility of the eye, which makes it suitable for intravitreal or subretinal injection surgery. AAVs can infect human cells, allowing long-term expression of the transgene after a single dose. Delivery of AAV by intravitreal injections is a safe method, but it often leads to low transduction efficiencies of PRs. A novel injection system using peripapillary intravitreal injection promises to be a safe and efficient alternative to standard intravitreal injections [153]. This, together with recent positive results on retinal-transduction efficiency of newly designed second-generation AAVs, combined with the accessibility of human retinal tissue through ROs to test their efficiency, holds hope for the future of AAV-based retinal therapies when combined with intravitreal delivery. 

Another type of therapeutic strategies are RNA-based therapies, such as antisense oligonucleotides (AONs), which are becoming popular in treating IRDs [154,155]. AONs are relatively small nucleic acid molecules that target the pre-mRNA or mRNA to modify the splicing process, alter translation, or degrade a transcript. Combining organoid applications with other technologies increases their considerable potential in clinical and translational research. CRISPR-Cas9 genome-editing technology, for example, may be employed virtually to modify PSC lines in order to generate ROs with any desired genetic modification [156,157]. As a genome-editable system, ROs are perfectly suitable to take advantage of the increasingly sophisticated toolbox being developed by optogeneticists, synthetic biologists, and even sonogeneticists to engineer new circuits and functions with remote switches in order to sensibly control cellular behavior [158,159,160].

### 7.2. Retinal Organoids as Human In Vitro Models

ROs open up a slew of new research opportunities, filling a gap left by inconsistencies between animal models and human disorders. The organoid cell population and the molecular profile appear to be similar to those of the human retina [161]. Recently, ROs have been employed to replicate IRDs [12,162], although organoids appear to be best suited to represent severe phenotypes with early disease onset. As previously reviewed by Zhang et al., 24 studies have so far modelled several aspects of retinal diseases using 3D ROs either derived from patients or from genetically manipulated PSCs. These diseases include retinitis pigmentosa, Leber’s congenital amaurosis, glaucoma, macular telangiectasia type 2, microphtalmia, retinoblastoma, Stargardt disease, and X-linked juvenile retinoschisis [163].

Other situations in which human PSC-derived retinas can be applied include screening for drugs that are active in a patient-specific genetic background. The PSC-derived retinas can be applied to the design of ‘‘clinical trials in a dish’’ by facilitating the sampling of a diverse population in drug-screening assays. In vitro ROs can be employed as testbeds to assess the pharmacological effects of moxifloxacin (a retinotoxic drug at higher dosages), resulting in effective reproduction of in vivo-like retinal cell damage, including the loss of PRs and amacrine cells [100]. Finally, ROs can be applied not only to the screening of compounds and small molecules, but also to the testing of gene therapy strategies and certain drug delivery approaches, including the use of nanoparticles.

### 7.3. Retinal Organoids as In Vitro Models for Retinal Prostheses

Despite the advancement in both research fields, ROs and retinal prostheses were always studied independently and in parallel. Surprisingly, we were unable to find any publication that directly intersected both fields. As mentioned earlier in this review, ROs can now represent key features of the native human retina and are currently used as disease models to decipher specific disease mechanisms to treat or reverse retinal degeneration [12,163]. In addition, few studies employed electrode arrays to show that hiPSCs-derived ROs are light-responsive [92,100], proving the applicability of ROs as in vitro models. However, to test the efficiency of an inorganic or organic retinal prosthesis and to quantify stimulated retinal activity, most of the recent studies have based their investigations on animal models of RDs or animal in vitro explants (Table 3). The retina in these models largely differ from the human retina and do not fully represent or recapitulate the human eye (patho)physiology [42,45]. Therefore, it is now of the utmost importance to use the potential of ROs, not only to investigate retinal disease mechanisms or gene- and cell-replacement therapies, but also as human in vitro models for retinal prosthesis optimization. The human organoid models can mimic the complex physiology of the retina in a more simplified setting, and therefore offer a more selective stimulation of retinal cell population. In addition, the identification and quantification of the photostimulated retinal activity of organic semiconductors in different configurations can be evaluated on the retinal tissue of human organoids derived from RD patients, instead of using ex vivo retina from animal models.

## 8. Conclusions

RDs are among the most complicated degenerative diseases because of the involvement of different factors, including multigenetic mutations, environmental factors, inflammatory processes, and ageing. With the discovery of iPSCs and, subsequently, the breakthroughs in their differentiation into 3D organoids, a new horizon of human in vitro models arose, including models of the retina. ROs, as humanized in vitro models, bridge the gap between animal experimental models and 2D cultures, and open various possibilities for understanding disease mechanisms in order to develop appropriate therapies. Many current RO protocols succeeded in recapitulating various aspects of the human eye retina, including a proper layer stratification and the presence of light sensitive PRs with outer segments and connecting cilia. 

Electronic retinal prostheses provide a concrete and proven solution in partially restoring vision for patients with late-onset RDs; some were FDA- and CE-approved and commercially available, but the manufacture was subsequently suspended due to poor visual resolution. Organic-based retinal prostheses promise an adequate alternative to classic silicon-based devices and are recently being extensively investigated. So far, only animal models of RDs are being used to validate their efficiency. Interestingly, ROs derived from human iPSC patients might provide a more humanized in vitro platform to investigate and improve the development of such emerging devices. Surprisingly, this area has not previously been explored, but we are currently investigating the feasibility of such ROs in our laboratory.

## Figures and Tables

**Table 1:** Most-used rodent models of retinal dystrophy. KO: knock-out; KI: knock-in; RCS: the Royal College of Surgeons.

Disease	Suitable Species	Rodent Models
Diabetic Retinopathy	Mice	Ins2Akita nonobese diabetic (*NOD*) ob/ob or Db/Db (*Lepr^db^*) Kimba; Akimba
Age-related Macular Degeneration	Mice, Rats, Rabbits; Pigs; Non-human primates	Complements *H, C3a* and *C5a* KO *C3* overexpression Chemokines *Ccl2/Cx3cr1* double KO *Nrf-2* or *SOD1* KO *ApoE4* KI
Glaucoma	Mice	TDBA/2J
Retinitis Pigmentosa	Mice; Rats; Rabbits; Pigs; Zebrafish; Non-human primates	rd1, rd4, rd8, rd10 VPP (*V20G*, *P27L*, *P23H* mutations) RCS rat model

**Table 2:** Summary of the key protocols developed to differentiate pluripotent stem cells to three- dimensional (3D) retinal organoids: hiPSC (human induced pluripotent stem cells); ESCs (embryonic stem cells); PR (photoreceptors); NR (neural retina); NRVs (neuroretinal vesicles); RPE (retinal pigment epithelium); EB (embryoid bodies); KSR (knock-out serum replacement); CC (connecting cilium); OV (optical vesicle); OC (optic cups); OF (optic fibers); d (days); w (weeks); m (months). Key factors in bold.

Study	Cell Source	Culture Initiation	Culture Diff.	Prot. Length	Tissues Produced	Notes
Lamba et al., 2006 [62]	hiPSCs	Matrigel-coated dishes. **noggin**, **DKK1**, **IGF1** for 3 w.	Cells cultured in N2/B27 medium	60 d	NR by d2; OC by d25; Rods PRs and OF by 6 w	First 2D retinal cells from ESCs
Nakano et al., 2012 [66]	hESCs	Matrigel KSR medium + **IWR-1e**, **ROCKi** for 12 d.	FBS, SAG for 6 d, DMEM/F12+N2 medium **Chir99021** d15 to d18 NR isolated on d18 in suspension culture.	126 d	Bi-layered OC of NR and RPE; PRs d126	3D method improvement
Phillips et al., 2012 [71]	Blood-derived hiPSCs	Cell aggregates in KSR for 4 d. N2 + **heparin** for 2 d.	Aggregates on laminin 10 d. d16, neural clusters in B27 medium. d20, OVs maintained in adherent culture.	50 d	OVs by d20; NR or RPE d40; NR rosettes d50	
Zhong et al., 2014 [67]	hiPSCs	Cell aggregates in mTeSR1 medium with **blebbistatin**. Medium gradually transitioned into N2 + heparin.	d16, B27 medium. w4, aggregates detached. d42, medium with FBS, **taurine**. Addition of **RA** for PR maturation.	21 w	NR; 3D retinal cups on d21–28; rhodopsin+ PRs by w21.	First 3D/2D method to describe mature and light-responding PRs
Reichman et al., 2014 [72]	hiPSCs	Confluent culture without FGF2 for 2 d, Medium transitioned into N2.	d14, neural clusters floating in N2 + **FGF2**; pigmented patches isolated on gelatin. FGF2 removed at d21.	30 d	Rapid diff. of NR and RPE; NR rosettes d42	First 2D/3D method; NRV excision
Zhou et al., 2015 [70]	hESCs hiPSCs	EBs cultured in KSR + B27, **noggin**, **DKK1**, **IGF1** for 3 d.	Adherent culture in N2/B27 + noggin, DKK1, IGF1, **COCO**, **FGF2** 4 w.	5 w	Cones PR d35; polarized cone PRs + CC + OS d60	
Singh et al., 2015 [73]	hESCs (H9)	Dense colonies in mTeSR1 + FGF2. Medium changed to FGF2- free Neurobasal medium + **noggin**. d3, N2/B27 added.	2 w + FGF2, 4 w **DKK1**+ **IGF1** for 1 w. Neurobasal medium + **noggin**, **FGF2**, **FGF9** for 12 w.	12 w	Four retina layers: RPE, early PRs, INL and RGCs	
Lowe et al., 2016 [74]	hESCs hiPSCs	Cell gelling for 30 min Floating clusters in N2/B27 medium 5 d.	d12–17, detach adherent cultures; floating aggregates in B27; 2 w + FBS + **taurine**	25–30 d	NR, ciliary margin, and RPE.	Spontaneous formationof NR
Völkner et al., 2016 [75]	hESCs	ROs in KSR + **ROCKi** + **IWR1e** 12 d, + Matrigel + FBS + SAG	ROs cut into 5 parts in N2 + FBS + **EC23** 41 d.	41 d	cone or rod PRs.	
Hunt et al., 2017 [76]	hESCs hiPSCs	EBs in mTeSR1 + **ROCKi**.	d3, KSR + **IGF1** + B27; d5–9, + FBS d12, EBs encapsulated in hydrogel 45 d.	45 d	NR and RPE	
Capowski et al., 2019 [77]	hESCs hiPSCs	hPSCs in mTeSR1 + Matrigel. EBs lifted and weaned in N2 + Heparin 4 d.	d6, + **BMP4**, d16, B27 + FBS + **taurine** + **RA**; d100. RA removed	175 d	Highly developed ONL, OPL, INL	staging system of ROs. BMP4 increases NRV
Kaya et al., 2019 [78]	hESCs hiPSCs	Cells in E8 + Matrigel. EBs lifted and weaned in N2 + Heparin 16 d.	d16, B27 + FBS; d42, + **taurine**; d63, + **9-cis retinal**; d92, half conc. + N2.	200 d	NR and cone, rod PRs	9-cis retinal enhance rod PRs.
Zerti et al., 2021 [79]	hESCs	Cells in mTeSR1 + Matrigel + ROCKi; KOSR + B27 + IGF-1 18 d.	d18, +**RA** + **IGF-1** + **T3** + **Taurine**; d37, N2/B27 + IGF-1	90 d	NR, RPE, lensand cornea; PRs by d90	IGF-1 increases the formation of laminated NRVs.
